# E-cadherin and β-catenin expression in early stage cervical carcinoma: a tissue microarray study of 147 cases

**DOI:** 10.1186/1477-7819-3-38

**Published:** 2005-06-21

**Authors:** Oluwole Fadare, Harini Reddy, Jun Wang, Denise Hileeto, Peter E Schwartz, Wenxin Zheng

**Affiliations:** 1 Department of Pathology, Yale University School of Medicine, New Haven, CT, USA; 2 Department of Obstetrics, Gynecology and Reproductive Sciences, Yale University School of Medicine, New Haven, CT, USA; 3 Department of Preventive Medicine, Keck School of Medicine, University of Southern California, Los Angeles, CA, USA

## Abstract

**Background:**

The disruption of intercellular adhesions is an important component of the acquisition of invasive properties in epithelial malignancies. Alterations in the cell-cell adhesion complex, E-Cadherin/β-Catenin, have been implicated in the oncogenesis of carcinomas arising from various anatomic sites and have been correlated with adverse clinico-pathologic parameters. In this study, the authors investigated the immunohistochemical expression of E-Cadherin and β-Catenin in a cohort of early stage cervical cancers to determine its prognostic significance and to investigate differences between the three major histological subtypes.

**Patients and methods:**

A tissue microarray of 147 cases of FIGO stage 1A and 1B cervical carcinomas [96 squamous cell carcinomas (SCC), 35 adenocarcinomas (AC), 12 adenosquamous carcinomas (ASQ), 4 miscellaneous types] was constructed from our archived surgical pathology files and stained with monoclonal antibodies to E-Cadherin and β-Catenin. Cases were scored by multiplying the intensity of staining (1 to 3 scale) by the percentage of cells stained (0–100%) for a potential maximum score of 300. For both markers, "preserved" expression was defined as bright membranous staining with a score of 200 or above. "Impaired" expression included any of the following: negative staining, a score less than 200, or exclusively cytoplasmic or nuclear delocalization.

**Results:**

Impaired expression of β-Catenin was found in 85.7%, 66.7%, & 58.3% of AC, SCC & ASQ respectively. Impaired expression of E-Cadherin was found in 94.3%, 86.5% & 100% of cases of AC, SCC, & ASQ respectively. The differences between the histologic subtypes were not significant. For the whole cohort, a comparsion of cases showing impaired versus preserved of E-Cadherin and β-Catenin expression showed no significant differences with respect to recurrence free survival, overall survival, patient age, histologic grade, and frequency of lymphovascular invasion or lymph node involvement. There was no correlation between the status of both markers for all three histological subtypes (overall spearman correlation co-efficient r = 0.12, p = 0.14)

**Conclusion:**

Impairment of E-Cadherin and β-Catenin expression is very frequent in early stage cervical cancers, and alterations in the E-Cadherin/β-Catenin cell adhesion complex are therefore likely involved in the pathogenesis of cervical carcinomas even at their earliest stages. None of the three major histological subtypes of cervical carcinoma (SCC, ADCA, ADSQ) is significantly more likely than the others to show impairment in E-Cadherin and β-Catenin expression. Overall, the expression of both markers does not significantly correlate with clinico-pathological parameters of prognostic significance.

## Background

The resounding success of the routine papanicolaou smear in reducing the incidence and mortality of cervical cancer has been chronicled extensively [1-6]. However, epidemiological data from 2004 indicates that 10,520 new cases of invasive cervical cancer are still diagnosed annually in the United States, with an associated mortality rate of approximately 37% [7]. For those patients with early stage (FIGO stage I and II) cervical cancers treated primarily with surgical therapy, the decision to administer adjuvant therapy is dependent on a variety of clinical and histopathological parameters. The latter includes the presence or absence of lymphovascular, or deep stromal invasion, large tumor size, tumor involvement of resection margins, involvement of regional lymph nodes etc [8]. Although histopathological parameters classify these patients into broad groups – each comprised of patients with similar risk-profiles for recurrence, the groupings are inherently non-specific [9]. Therefore, it is anticipated that some patients with cervical cancer receive unnecessary adjuvant therapy, with their attendant toxicities. It would thus be of interest to identify biomarkers which can be evaluated on the primary tumor and may further help refine the subset of patients at the most significant risk for recurrence and thus in need of intensive adjuvant management.

The disruption of intercellular adhesions is an important component of the acquisition of invasive properties in epithelial malignancies. Alterations in the cell-cell adhesion complex, E-Cadherin/β-Catenin, have been implicated in the oncogenesis of carcinomas arising from various anatomic sites and have been correlated with adverse clinico-pathological parameters [10-22]. Epithelial Cadherin (E-Cadherin) is a 120 kDa transmembrane glycoprotein which is involved in both homotypic and heterotypic Ca2^+^-dependent cellular adhesions [23-26]. Inactivation of E-Cadherin may occur through mutations, methylations or deletions of the E-cadherin gene, suppression of the E-Cadherin gene promoter, or posttranslational modification of the protein leading to cytoplasmic delocalization [27-30]. The strength of E-Cadherin-mediated intercellular adhesion is significantly increased by interactions between the cytoplasmic tail of E-cadherin and the cytoskeletal network [30]. This interaction is mediated through the cytoplasmic proteins β-Catenin, α-Catenin and γ-Catenin [23-26]. β-Catenin is a 92 kDa protein that, in addition to its cell-adhesion properties, alsoplays a role as a transcriptional co-activator in the *Wnt *signaling pathway; the latter is involved in cellular development, differentiation and oncogenesis [32,33]. Deregulation of the *Wnt *pathway may occur through an activating mutation of the β-Catenin gene, leading to accumulated levels of β-Catenin in the cytoplasm and nucleus, and culminating in the altered transcription of a variety of critical genes [26].

The cervix, in which a dysplasia-to-carcinoma sequence is well-established, offers a useful medium to comparatively study the expression of proteins involved in cell-to-cell adhesion in dysplastic and invasive epithelium. Previous studies have shown that the expression of E-Cadherin and β-Catenin, as evaluated immunohistochemically, is inversely proportional to the histologic grade in squamous intraepithelial lesions (SIL) of the cervix: expression of both markers is generally maintained in low-grade lesions and is lost in high grade lesions [34-36]. In the present study, the expression of E-Cadherin and β-Catenin was evaluated on a cohort of already invasive, albeit early stage cervical carcinomas. Our objectives are 1) to determine the frequency of loss of expression of E-Cadherin and β-Catenin in early stage cervical carcinomas 2) to determine whether the expression of either of these biomarkers is of prognostic significance 3) to specifically investigate whether there are any differences between the three major histological subtypes of cervical carcinoma with respect to expression of E-Cadherin and β-Catenin.

## Patients and methods

### Case selections and microarray construction

Following approval from our institutional review board, a tissue microarray (TMA) [37] of 147 cases of international federation of gynecology and obstetrics (FIGO) stage 1a and 1b cervical carcinomas was constructed. Technical details on microarray construction at the Yale tissue microarray facility are outlined elsewhere [38,39]. The cases were retrieved from the archived surgical pathology files at Yale New Haven Hospital and consisted of carcinomas diagnosed between 1987 and 2001. These cases were not consecutive but were selected on the basis of availability of at least one evaluable tumor-representative hematoxylin and eosin-stained slide and a paraffin block. On each case, slides were reviewed to select a representative tumor spot. The corresponding spot on the associated paraffin block was then cored and placed on a tissue microarrayer (Beecher Instruments, Silver Spring, MD, USA). The microarray was constructed with a 2-fold redundancy (2 spots for each patient), which has been previously shown by Camp *et al *[40] to accurately represent standard tissue sections in at least 95% of cases. The finalized arrays were then cut into 5 μm-thick sections and mounted on glass slides using an adhesive tape-transfer system (Instrumedics Inc., Hackensack, NJ, USA) with ultraviolet cross-linking.

### Normal controls

To evaluate the staining patterns of E-Cadherin and β-Catenin in the normal cervix, 5 such cases were retrieved from our database. These 5 cases were hysterectomies performed for adnexal masses in which the final diagnoses were non-neoplastic (endometriosis [n = 4], tubo-ovarian abscess [n = 1]), Sections from the cervix in these cases were then stained with monoclonal antibodies to E-Cadherin and β-Catenin.

### Immunohistochemistry

The tissue microarray slides were stained with antibodies to E-Cadherin (Clone NCH-38, dilution 1:100, DakoCytomation Corporation, Carpinteria, CA, USA) and β-Catenin (Clone 17C2, dilution 1:100, NovoCastra Laboratories Ltd., Tyne, UK) using a DAKO autostainer based on the standard avidin-biotin complex method. Tissue microarray slides were deparaffinized with xylene and graded alcohols then rehydrated with distilled water. Endogenous peroxidase activity was blocked by placing the slides in 0.5% hydrogen peroxidase/methanol for 10 minutes followed by a tap water rinse. Background staining was reduced by incubating slides in 0.3% bovine serum albumin/Tris-buffered saline. Antigen retrieval entailed placing the slides in a pressure cooker with an antigen unmasking solution (0.01M citrate buffer, pH 6.0) for 1 minute. Slides were subsequently incubated with the primary (4°C overnight), then biotinylated secondary antibodies and streptavidin-biotin peroxidase. 0.05% 3'3' diaminobenzidine (DAB) was used as chromogen, followed by counterstaining with hematoxylin.

### Scoring of immunohistochemical staining

**Figure 1:**
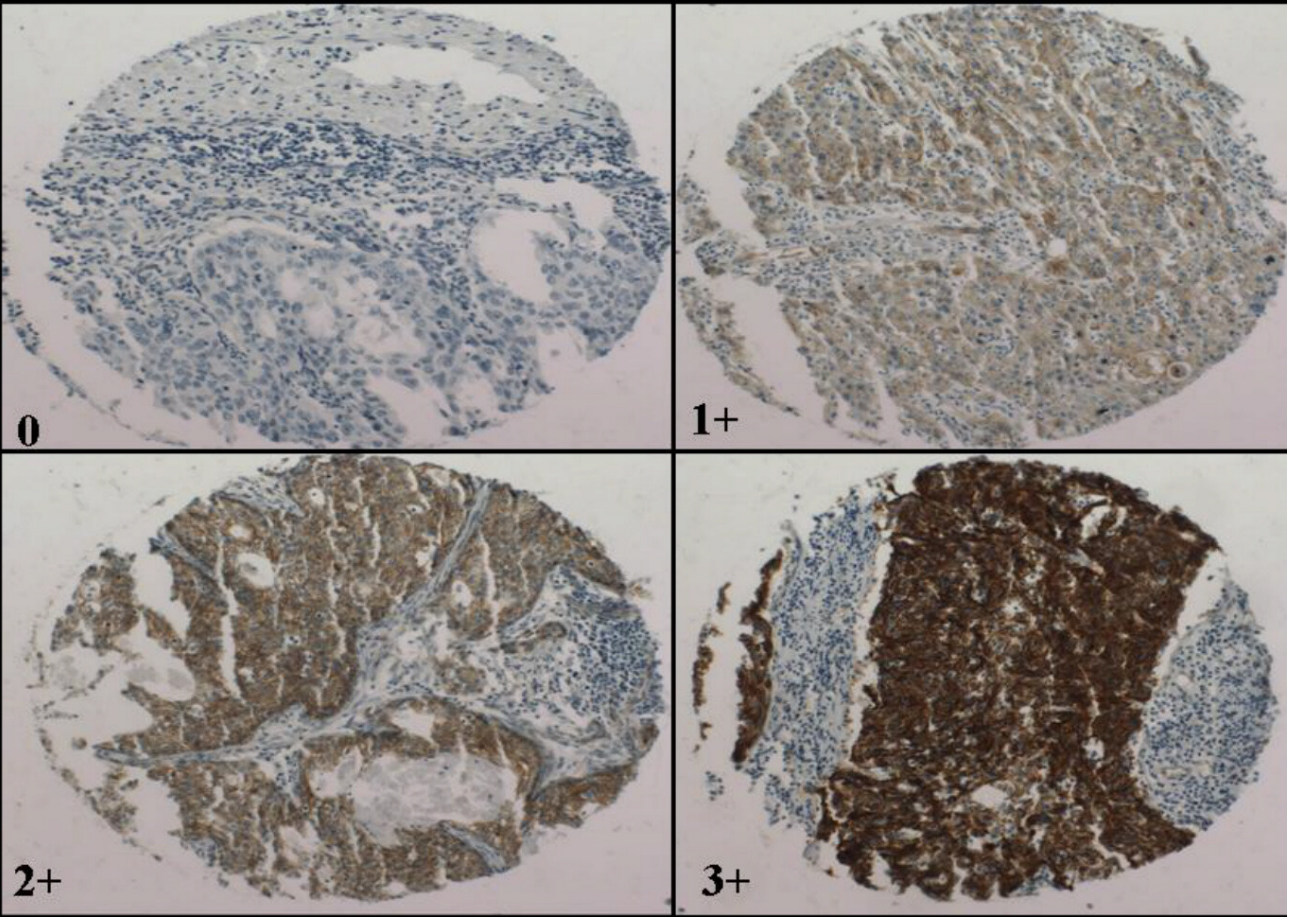
Representative cores illustrating the criteria used for scoring staining intensity (β-Catenin is shown; E-Cadherin was similar, although with an overall lower intensity).

Since both E-Cadherin and β-Catenin are normally expressed in a bright membranous fashion in the cervical epithelium, aberrations on this basic theme were deemed abnormal for the purposes of this study. Thus, cytoplasmic staining, nuclear staining and no staining were considered "impaired" expression. Cases were scored by multiplying the intensity of staining (1 to 3 scale, see figure 1) by the percentage of cells stained (0–100%) for a potential maximum score of 300. For both markers, "preserved" expression was defined as bright membranous staining with a score of 200 or above. "Impaired" expression included any of the following: negative staining, a score less than 200, or exclusively cytoplasmic and/or nuclear delocalization. Only the core showing the highest score was utilized in each case. In 144 of 147 cases, the scores did not differ between the 2 cores on each case by more than 50 points. None of the 3 cases with such a discrepancy (i.e. differences of more than 100 points between the 2 cores) affected the "impaired" versus "preserved" expression distinction, because all 3 cases showed impaired expression in both cores. The microarrays were scored by 2 independent observers (OF and DH). Discrepancies were resolved at a consensus session. Results were then correlated with a variety of pathologic parameters, including tumor size, frequency of lymphovascular invasion, lymph node involvement and histological grade.

### Statistical analysis

For comparison of cases showing impaired versus preserved expression with respect to various clinico-pathological parameters, two-sided likelihood ratio chi-square test or Fisher's exact tests were used with p < 0.05 as the "statistically significant" cut-off point. Survival analysis was carried out by Kaplan-Meier method and Wilcoxon test was used to compare survival. Spearman correlation coefficients were calculated to examine the correlations between the two markers. All analyses were performed using SAS version 9.0 (SAS Institute, NC).

### Validation

To externally validate the staining patterns observed on the microarray, 14 cases representing approximately 10% of the array were selected in a semi-random fashion using a randomization function (Microsoft Excel^®^, Microsoft Inc, Redmond, WA, USA) on a list of the entire cohort. The goal was to obtain a representative mix of cases with negative and positive stainings (4 groups, each consisting of 3 cases with a score of 0, 1–99, 100–199, 200–300; 2 additional cases for the last group). Thus, each case selected from the list by the randomization function was included in the validation list consecutively until the preset quantitative requirement for that group was met. On each of these 14 cases, a full representative tissue section was then stained with E-Cadherin and β-Catenin and scored using the same system used with the microarray.

## Results

### Normal epithelium

**Figure 2:**
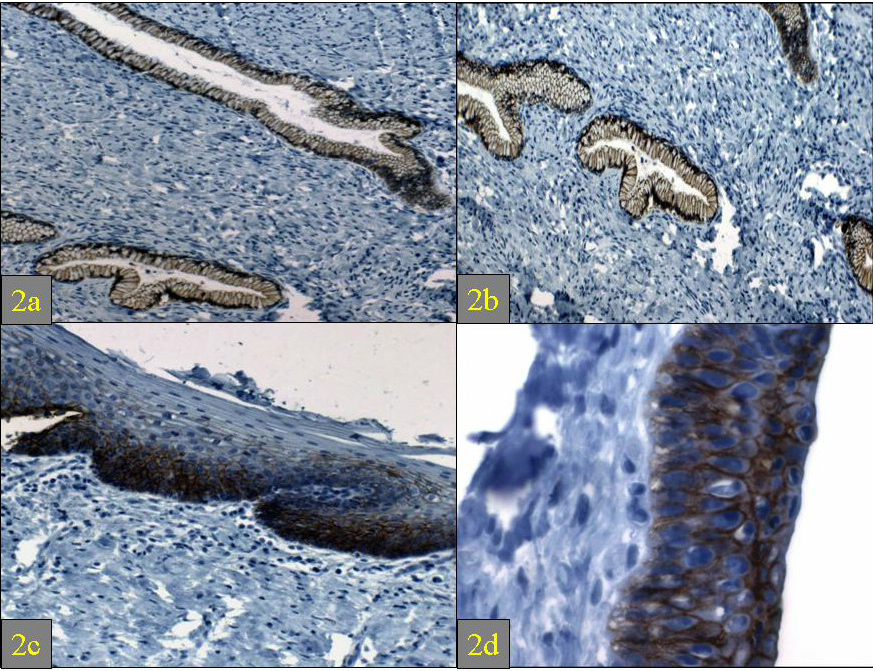
Expression of E-Cadherin in the normal endocervix (2a, hematoxylin and eosin, original magnification ×100; 2b: hematoxylin and eosin, original magnification ×200), ectocervix (2c: hematoxylin and eosin, original magnification ×100) and squamous metaplastic epithelium at the transformation zone (2d) (hematoxylin and eosin, original magnification ×200).

The staining patterns of E-Cadherin and β-Catenin were identical. However, β-Catenin staining was generally of greater intensity than E-Cadherin staining. In the endocervices of all 5 cases, both markers brightly decorated the epithelium in a circumferentially membranous fashion (basolateral and glycocalyceal). No cytoplasmic or nuclear staining was noted (Figures 2a and 2b). In the ectocervix, staining was also membranous but was limited to the lower two-thirds in 4 cases and the lower half in 1 case (Figure 2c). Squamous metaplastic epithelium was present in 3 cases, all of which showed transmural bright membranous staining (Figure 2d). In all 5 cases, the basal and parabasal cells displayed the greatest intensity of staining (3/3). There was no stromal staining.

### Tissue microarray

The 147 cases consisted of 96 squamous cell carcinomas (SCC), 35 adenocarcinomas (ADCA), 12 adenosquamous carcinomas (ADSQ) and a heterogeneous group of 4 cases hereafter referred to as "others". The latter group included 1 small cell carcinoma, 1 pure large cell neuroendocrine carcinoma, 1 squamotransitional carcinoma and 1 adenosquamous carcinoma with morphologically-evident neuroendocrine differentiation. 4 cases were deemed unsatisfactory during scoring by virtue of poor tissue preservation on the microarray slides. The clinicopathological characteristics of the cohort are outlined in table 1.

**Table 1:** Summary of Clinical and Pathological characteristics of Cohort

**Clinicopathologic Parameter**		**n**
**Age (years)**	Median	40
	Mean	42
	Range	20–77
**FIGO stage**	1A1	14
	1A2	15
	1B1	109
	1B2	9
**Lymphovascular invasion**	Present	62
	Absent	85
**Histological grade**	1	33
	2	54
	3	60
**Lymph node involvement**	Present	18
	Absent	129
**Tumor Size (cm)**	0.1–0.5	44
	0.6–1.0	28
	1.1–2.0	33
	2.1–3.0	21
	>3.1	21
**Follow-up (years)**	Median	4.75
	Mean	5.84
	Range	0.12–17.43

FIGO: International Federation of Gynecology and Obstetrics

#### β-Catenin

**Figure 3:**
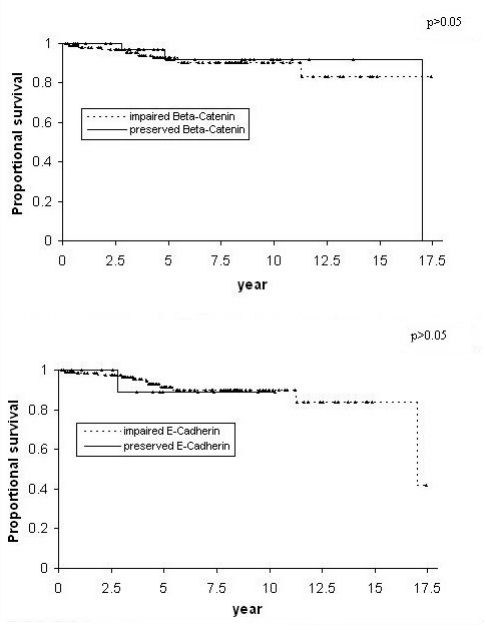
Kaplan-Meier recurrence-free survival curves comparing patients whose cervical carcinomas showed preserved expression of E-Cadherin and β-Catenin to those with impaired expression (▲ indicates censored observations).

**Figure 4:**
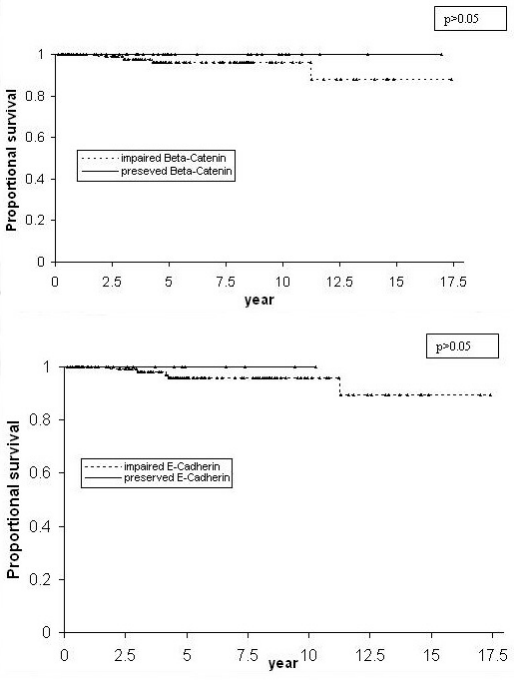
Kaplan-Meier overall survival curves comparing patients whose cervical carcinomas showed preserved expression of E-Cadherin and β-Catenin to those with impaired expression (▲ indicates censored observations).

Impaired expression of β-Catenin was present in 107 (75%) of the 143 cases and in the majority of all the histological subtypes. ADCA had the highest percentage of impaired beta-catenin expression (85.7%, 30/35). Compared to ADCA, a smaller proportion of cases of SCC showed impaired beta-catenin expression (66.7%, 64/96). However, the difference was not significant (p = 0.06). ADSQ showed an even lower proportion of cases with impaired beta-catenin expression (58.3%, 7/12). Compared to ADCA, the difference was not significant (p = 0.06). The difference between SCC and ADSQ was also not significant (p = 0.47). Impairment of expression was most commonly in the form of absent staining (79/107); 19 cases did not meet out quantitative criteria for positivity while 5 cases showed exclusively cytoplasmic delocalization. There were no cases of exclusively nuclear staining (Table 2). The mean age for the patients with preserved and impaired β-Catenin expression was 41.5 years (± 10.6) (n = 39) and 41.6 years (± 11.5) (n = 104) respectively. There was no significant difference (p = 0.99) between these 2 groups. Similarly, there were no statistically significant differences with respect to recurrence free survival (Figure 3), overall survival (Figure 4), frequency of lymphovascular invasion, histologic grade, and frequency of lymph node involvement (Table 3) when cases with impaired expression of β-Catenin were compared with those in which β-Catenin was preserved.

**Table 2:** Number of patients expressing E-Cadherin and β-Catenin among the various histologic subtypes

**Histologic subtype of cervical carcinoma**	**Preserved^¶^**	**Impaired**				**U**
			
		**Reduced Staining **▼	**Exclu-Sively Cyto-Plasmic Stain Ing**	**Negative Staining**	**Nuclear Staining**	
**β-catenin expression**						

**Squamous cell Carcinoma (n = 96)**	29	10	2	52	0	3
**Adenocarcinoma (n = 35)**	5	7	1	22	0	0
**Adenosquamous (n = 12)**	5	1	2	4	0	0
**Others (n = 4)**	1	1	0	1	0	1
**Total (n = 147)**	**40**	**19**	**5**	**79**	**0**	**4**

**E-cadherin expression**						

**Squamous cell Carcinoma (n = 96)**	11	16	2	65	0	2
**Adenocarcinoma (n = 35)**	1	5	3	25	0	1
**Adenosquamous (n = 12)**	0	3	2	7	0	0
**Others (n = 4)**	1	0	1	2	0	0
**Total (n = 147)**	**13**	**24**	**8**	**98**	**0**	**4**

U: unsatisfactory

¶ membranous staining with 200 score or higher

▼less than 200 score

**Table 3:** Lack of association between expression of E-Cadherin or β-Catenin and lymphovascular invasion, frequency of lymph node involvement or histologic grade

**Preserved^¶^**		***Impaired***				
			
		**Reduced Staining **▼	**Exclusively Cytoplasmic Staining**	**Negative Staining**	**Nuclear Staining**	**p value (impaired versus preserved expression)**
**1) Lymphovascular invasion**						

**β-Catenin expression**						

Absent (n = 83)	20	7	2	54	0	0.4
Present (n = 60)	20	12	3	25	0	
**E-Cadherin expression**						
			
Absent (n = 83)	6	11	3	63	0	0.4
Present (n = 60)	7	13	5	35	0	
2) Histological grade*						
**β-Catenin expression**						

1 (n = 31)	8	3	1	19	0	>0.05 for all comparisons
2 (n = 53)	20	7	1	25	0	
3 (n = 59)	12	9	3	35	0	
**E-Cadherin expression**						
1 (n = 31)	1	5	2	23	0	>0.05 for all comparisons
2 (n = 53)	4	13	3	33	0	
3 (n = 59)	8	6	3	42	0	
**3) Lymph node Involvement**						
**β-Catenin expression**						

Absent (n = 125)	33	15	5	72	0	0.3
Present (n = 18)	7	4	0	7	0	
**E-Cadherin expression**						
			
Absent (n = 125)	9	21	7	88	0	0.06
Present (n = 18)	4	3	1	10	0	

¶ membranous staining with 200 score or higher

▼less than 200 score

#### E-Cadherin

Of the 143 cases, 134 (94%) showed impaired expression of E-Cadherin. Notably, impaired expression of E-Cadherin was present in 100% of the 12 ADSQ; however, high frequencies of impaired expression were also present in ADCA and SCC and the differences between the histologic subtypes were not statistically significant. Impairment of expression was most commonly in the form of absent staining (99/134); 24 cases did not meet out quantitative criteria for positivity while 8 cases showed exclusively cytoplasmic delocalization. No case showed nuclear staining (Table 2). The mean age for the patients with preserved and impaired E-Cadherin expression was 36.5 years (± 9.1) (n = 12) and 42.4 years (± 11.7) (n = 131) respectively. Thus, patients with impaired E-Cadherin expression appeared to be older than patients with preserved E-Cadherin expression. However, the difference was not statistically significant (p = 0.09). As with β-Catenin, there were no statistically significant differences with respect to recurrence free survival (Figure 3), overall survival (Figure 4), frequency of lymphovascular invasion, lymph node involvement and histologic grade (Table 3) when cases with impaired expression of E-Cadherin were compared with those in which E-Cadherin was preserved.

#### β-Catenin and E-Cadherin correlation analysis

Only six (4%) of 143 cases showed preservation of *both *E-Cadherin and β-Catenin expression and 93 (65%) of 143 cases showed impairment in the expression of both markers, 7 cases showed impairment in β-Catenin and preservation of E-Cadherin whereas the reverse was observed in 34 cases. Overall, the expressions of E-Cadherin and β-Catenin were not significantly correlated (spearman correlation coefficient r = 0.12, p = 0.14). Similar findings were noted when each of the 3 major histological subtypes was subjected to the same analysis (table 4)

**Table 4:** Lack of correlation between E-Cadherin and β-Catenin expression among the 3 major histologic subtypes of invasive carcinoma

	**Histological Subtype**		**E-Cadherin expression**		**Correlation**
				
			**Preserved**	**Impaired**	
**β-Catenin expression**	**Squamous cell carcinoma**	**Preserved**	5	24	
		**Impaired**	6	56	r = 0.11 p = 0.3
	**Adenocarcinoma**	**Preserved**	0	5	
		**Impaired**	1	28	r = -0.07 p = 0.7
	**Adenosquamous carcinoma**	**Preserved**	0	5	
		**Impaired**	0	7	Not applicable

#### Validation

In 13 of 14 cases, the scores obtained on standard histologic sections were within 50 points of the scores obtained on the corresponding tissue microarray spot (average difference: 30). In the 14th case, the tissue microarray spot was scored 0 whereas the corresponding histologic section was scored 100. In none of the 14 cases did a score discrepancy affect the "preserved" versus "impaired" distinction.

## Discussion

In this study, we demonstrate that 1) loss of expression of both E-Cadherin and β-Catenin are frequent events in early stage cervical carcinomas of all three major histological subtypes, and both proteins are thus likely participants in the pathogenesis of cervical carcinoma, 2) Loss of expression of both proteins is of no prognostic significance with respect to the following parameters: recurrence free survival, overall survival, frequency of lymphovascular invasion, histologic grade, and frequency of lymph node involvement. Our findings are in concordance with the recent study of Van de Putte *et al *[41], in which cases of stage 1B SCC were analyzed. In that study, loss of expression of E-Cadherin and β-Catenin in greater than 50% of tumor cells was found in 90% and 72% of cases respectively. However, the authors found no correlation between the expression of E-Cadherin and β-Catenin (and other catenins) and prognosis [41]. In contrast, Jeffers *et al *[42] found strong expression of E-Cadherin in all 20 cases of invasive cervical carcinoma that were studied. However, those 20 cases were an admixture of all stages and the prognostic significance of E-Cadherin was not specifically investigated since all cases were positive. Other studies have also showed a high frequency of loss of E-Cadherin expression in cervical cancers. Applying the criteria of the present study, impaired expression of E-cadherin was found in 89% of invasive SCCs in one study [34]. In the study of Sun *et al *[43], 60 cases of invasive cervical carcinoma of all stages were investigated for E-cadherin expression. Loss of expression of E-Cadherin was found in 53.3% of cases. Additionally, the authors found a correlation between abnormal E-Cadherin expression and clinical stage and, in contrast to the present study, lymph node involvement and histologic grade. Similarly, among all stages of cervical adenocarcinoma, abnormal expression of β-Catenin was significantly associated with advanced pathologic stage and thus disease-free survival in another study [44]. In the latter two studies, the noted discrepancies with the current one may be related to qualitative technical differences (antibody concentration, antigen retrieval methods etc) or differences in interpretation (i.e. criteria for positivity). None of the above studies have specifically compared the 3 major histologic subtypes in early stage cervical carcinoma. However, the majority of studies show that loss of expression of E-Cadherin and β-Catenin is frequent in cervical carcinomas. Although their impact on prognosis of this expression is less certain, our study finds that the expression of both E-Cadherin and β-Catenin lacks prognostic significance at least in early stage cervical carcinoma.

An orderly, membranous expression of E-Cadherin and β-Catenin is found in the normal cervix. The loss of expression of both proteins in a high proportion of high-grade squamous intraepithelial lesions [34-36] suggests that dysregulation of this pathway is an early event in cervical carcinogenesis. We investigated cytoplasmic and nuclear staining as a manifestation of impaired expression of both E-Cadherin and β-Catenin [26]. In the typical normal cell β-Catenin's complex with E-Cadherin and the cytoskeletal network is inversely proportional to the association of β-Catenin with the adenomatous polyposis coli protein, a large multifunctional cytosolic protein. Thus, normally, only small portions of β-Catenin are found in the cytoplasm since the association of β-Catenin with APC eventuates in its lytic degradation. Cytoplasmic accumulation often leads to nuclear accumulation, where β-Catenin may interact with a variety of proteins, culminating in transcriptional activation of a variety of critical genes [23-26]. Experimental studies have shown that tyrosine phosphorylation of β-Catenin by oncogenic products or growth factor receptors may cause dissociation of the E-Cadherin-associated adhesion complex from the cytoskeleton resulting in cellular (non-membranous) redistribution and disassociation of adherens junctions, a well-known feature of epithelial malignancies [45,46]. Exclusively cytoplasmic localization of β-Catenin and E-Cadherin, which was not present in any of the normal cervix epithelia, was seen in 3.4% and 5.4% of our carcinoma cases respectively. A progressive increase in the proportion of cases showing this finding was found in low grade SIL, high grade SIL and invasive carcinomas in one study [34]. However, the logical extension of that finding, a correlation with histological grade in invasive carcinomas, was not found in this study. Our study is also in concordance with that of Shinohara *et al *[47], in which no relationship was found between the frequency of cytoplasmic/nuclear localization of β-Catenin and histologic grade (no cases of nuclear localization β-Catenin was found in our study).

The possibility that our findings may have been significantly affected by our methods cannot be entirely excluded. Tissue microarray technology as a high throughput modality has gained wide acceptance in pathology investigations and is used routinely [48-52]. Our TMA was constructed with a two-fold redundancy to minimize sampling errors [40]. Furthermore, in our validation set which constituted approximately 10% of the TMA, no significant discrepancies were seen. Nonetheless, error introduced by tumor heterogeneity remains a possibility. Most notably, the minimization of errors from the redundant (2-fold) construction of our TMA presumes a similar degree of tumor heterogeneity between breast [40] and cervical cancer, which may be untrue.

The importance of defining thresholds in any investigations reporting the loss of the immunohistochemical expression of a biomarker cannot be overemphasized. As previously noted, a threshold of 200 was used in this study because both E-Cadherin and β-Catenin are normally expressed in a membranous fashion in most of the cervical epithelium (scores 270–300 for the ectocervix and 300 for the endocervix). However, we performed separate statistical analyses when the threshold was lowered, i.e. when "impaired" expression included any of the following: negative staining, a score less than 99, or exclusively cytoplasmic and/or nuclear delocalization. The results on all previous analyses remained the same (p > 0.05), with 2 exceptions: 1) Overall, the expressions of E-Cadherin and β-Catenin became significantly correlated (spearman correlation coefficient r = 0.33, p = 0.0001). 2) 73% of LVI-negative carcinomas showed impaired β-Catenin expression, compared to 47% of LVI-positive carcinomas (p = 0.03). The significance of the latter finding is unclear, and it can be anticipated that changes in the threshold in either direction (lowering or raising) would result in at least one parameter attaining statistical significance with each change. In our opinion, for a marker that is normally expressed, a high threshold should be used for "loss" of expression, and 200/300 seems reasonable.

## Conclusion

In the present report we demonstrated that impairment of E-Cadherin and β-Catenin expression is very frequent in early stage cervical cancers. None of the three major histological subtypes of cervical carcinoma (SCC, ADCA, ADSQ) is significantly more likely than the others to show impairment in E-Cadherin and β-Catenin expression. Alterations in the E-Cadherin/β-Catenin cell adhesion complex are therefore likely involved in the pathogenesis of cervical carcinomas even at their earliest stages. However, the expression of both markers does not significantly correlate with clinicopathologic parameters of prognostic significance.

## Competing Interests

The author(s) declare that they have no competing interests.

## Authors' Contributions

**OF **wrote the initial version of the manuscript

**HR **collected clinical data and summarized clinicopathologic information

**JW **performed statistical analysis and helped summarize clinicopathologic information

**DH **and **OF **scored the tissue microarray

**WZ **and **PES **conceived of, supervised, and sponsored the project. Both revised the manuscript.

All authors have read and approved the final manuscript

## References

[B1] Koss LG (1989). The Papanicolaou test for cervical cancer detection: a triumph and tragedy. JAMA.

[B2] van de Graaf Y, Vooijs GP, Zielhuis GA (1990). Cervical screening revisited. Acta Cytol.

[B3] Devesa SS, Young JL, Brinton LA, Fraumeni JF (1989). Recent trends in cervix uteri cancer. Cancer.

[B4] Wingo PA, Cardinez CJ, Landis SH, Greenlee RT, Ries LA, Anderson RN, Thun MJ (2003). Long-term trends in cancer mortality in the United States, 1930–1998. Cancer.

[B5] Adami HO, Ponten J, Sparen P, Bergstrom R, Gustafsson L, Friberg LG (1994). Survival trend after invasive cervical cancer diagnosis in Sweden before and after cytologic screening, 1960–1984. Cancer.

[B6] Benedet JL, Anderson GH, Matistic JP (1992). A comprehensive program for cervical cancer detection and management. Am J Obstet Gynecol.

[B7] Jemal A, Tiwari RC, Murray T, Ghafoor A, Samuels A, Ward E, Feuer EJ, Thun MJ (2004). Cancer Statistics, 2004. CA Cancer J Clin.

[B8] Schilder JM, Stehman FB (2003). Stage Ia-IIa cancer of the cervix. Cancer J.

[B9] Sedlis A, Bundy BN, Rotman MZ, Lentz SS, Muderspach LI, Zaino RJ (1999). A randomized trial of pelvic radiation therapy versus no further therapy in selected patients with stage IB carcinoma of the cervix after radical hysterectomy and pelvic lymphadenectomy: A Gynecologic Oncology Group Study. Gynecol Oncol.

[B10] Shimazui T, Schalken JA, Giroldi LA, Jansen CF, Akaza H, Koiso K, Debruyne FM, Bringuier PP (1996). Prognostic value of cadherin-associated molecules (α-, β-, and γ-catenins and p120^cas^) in bladder tumors. Cancer Res.

[B11] Krishnadath KK, Tilanus HW, van Blankenstein M, Hop WC, Kremers ED, Dinjens WN, Bosman FT (1997). Reduced expression of the cadherin-catenin complex in esophageal adenocarcinoma correlates with poor prognosis. J Pathol.

[B12] van der Wurff AA, Vermeulen SJ, van der Linden EP, Mareel MM, Bosman FT, Arends JW (1997). Patterns of α-and β-catenin and E-cadherin expression incolorectal adenomas and carcinomas. J Pathol.

[B13] De Leeuw WJ, Berx G, Vos CB, Peterse JL, Van de Vijver MJ, Litvinov S, Van Roy F, Cornelisse CJ, Cleton-Jansen AM (1997). Simultaneous loss of E-cadherin and catenins in invasive lobular breast cancer and lobular carcinoma *in situ*. J Pathol.

[B14] Shimazui T, Bringuier PP, van Berkel H, Ruijter E, Akaza H, Debruyne FM, Oosterwijk E, Schalken JA (1997). Decreased expression of alpha-catenin is associated with poor prognosis of patients with localized renal cell carcinoma. Int J Cancer.

[B15] Zheng Z, Pan J, Chu B, Wong YC, Cheung AL, Tsao SW (1999). Down regulation and abnormal expression of E-cadherin and β-catenin in nasopharyngeal carcinoma: Close association with advanced disease stage and lymph node metastasis. Hum Pathol.

[B16] Perl AK, Wilgenbus P, Dahl U, Semb H, Christofori G (1998). A causal role for E-cadherin in the transition from adenoma to carcinoma. Nature.

[B17] Jawhari AU, Noda M, Farthing MJ, Pignatelli M (1999). Abnormal expression and function of the E-cadherin-catenin complex in gastric carcinoma cell lines. Br J Cancer.

[B18] Ross JS, Figge HL, Bui HX, del Rosario AD, Fisher HA, Nazeer T, Jennings TA, Ingle R, Kim DN (1994). E-cadherin expression in prostatic carcinoma biopsies: Correlation with tumor grade, DNA content, pathologic stage, and clinical outcome. Mod Pathol.

[B19] Umbas R, Isaacs WB, Bringuier PP, Schaafsma HE, Karthaus HF, Oosterhof GO, Debruyne FM, Schalken JA (1994). Decreased E-cadherin expression is associated with poor prognosis in patients with prostate cancer. Cancer Res.

[B20] Faleiro-Rodrigues C, Macedo-Pinto I, Pereira D, Lopes CS (2004). Loss of β-Catenin is associated with poor survival in ovarian carcinomas. Int J Gynecol Pathol.

[B21] Li Z, Ren Y, Lin SX, Liang YJ, Liang HZ (2004). Association of E-cadherin and beta-catenin with metastasis in nasopharyngeal carcinoma. Chin Med J.

[B22] Chang HJ, Jee CD, Kim WH (2002). Mutation and altered expression of beta-catenin during gallbladder carcinogenesis. Am J Surg Pathol.

[B23] Conacci-Sorrell M, Zhurinsky J, Ben-Ze'ev (2002). The cadherin-catenin adhesion system in signaling and cancer. J Clin Invest.

[B24] Gooding JM, Yap KL, Ikura M (2004). The cadherin-catenin complex as a focal point of cell adhesion and signaling: new insights from three-dimensional structures. BioEssays.

[B25] Nelson WJ, Nusse R (2004). Convergence of Wnt, β-Catenin, and Cadherin pathways. Science.

[B26] Hajra KM, Fearon ER (2002). Cadherin and Catenin alterations in human cancer. Genes, Chromosomes Cancer.

[B27] Berx G, Cleton-Jansen AM, Nollet F, de Leeuw WJ, van de Vijver M, Cornelisse C, van Roy F (1995). E-cadherin is a tumour/invasion suppressor gene mutated in human lobular breast cancers. EMBO J.

[B28] Vos CB, Cleton-Jansen AM, Berx G, de Leeuw WJ, ter Haar NT, van Roy F, Cornelisse CJ, Peterse JL, van de Vijver MJ (1997). E-cadherin inactivation in lobular carcinoma in situ of the breast: an early event in tumorigenesis. Br J Cancer.

[B29] Nawrocki B, Polette M, Van Hengel J, Tournier JM, Van Roy F, Birembault P (1998). Cytoplasmic redistribution of E-cadherin-catenin adhesion complex is associated with down-regulated tyrosine phosphorylation of E-cadherin in human bronchopulmonary carcinomas. Am J Pathol.

[B30] Hirohashi S (2000). Molecular aspects of adhesion-epigenetic mechanisms for inactivation of the E-Cadherin-mediated cell adhesion system in cancers. Verh Dtsch Ges Pathol.

[B31] Yap AS, Brieher Wm, Pruschy M, Grumbiner WM (1997). Lateral clustering of the adhesive ectodomain: a fundamental determinant of Cadherin function. Curr Biol.

[B32] Miller JR, Hocking AM, Brown JD, Moon RT (1999). Mechanism and function of signal transduction by the Wnt/beta-Catenin and Wnt/Ca^2+ ^pathways. Oncogene.

[B33] Peifer M, Polakis P (2000). Wnt signaling in oncogenesis and embryogenesis-a look outside the nucleus. Science.

[B34] Faleiro-Rodrigues C, Lopes C (2004). E-cadherin, CD44 and CD44v6 in squamous intraepithelial lesions and invasive carcinomas of the uterine cervix: an immunohistochemical study. Pathobiology.

[B35] de Boer CJ, van Dorst E, van Krieken H, Jansen-van Rhijn CM, Warnaar SO, Fleuren GJ, Litvinov SV (1999). Changing roles of cadherins and catenins during progression of squamous intraepithelial lesions in the uterine cervix. Am J Pathol.

[B36] Yang JZ, Zhang XH, Wu WX, Yan X, Liu YL, Wang JL, Wang FR (2003). Expression of EP-CAM, beta-catenin in the carcinogenesis of squamous cell carcinoma of uterine cervix. Zhonghua Zhong Liu Za Zhi.

[B37] Kononen J, Bubendorf L, Kallioniemi A, Barlund M, Schraml P, Leighton S, Torhorst J, Mihatsch MJ, Sauter G, Kallioniemi OP (1998). Tissue microarrays for high-throughput molecular profiling of tumor specimens. Nat Med.

[B38] Charette L, Rimm DL Yale tissue microarray construction protocols. Version 1.0, 1/2001. http://tissuearray.org/yale/tisarray.html. Accessed 02/20/05.

[B39] Rimm DL, Camp RL, Charette LA, Olsen DA, Provost E (2001). Amplification of tissue by construction of tissue microarrays. Exp Mol Pathol.

[B40] Camp RL, Charette LA, Rimm DL (2000). Validation of tissue microarray technology in breast carcinoma. Lab Invest.

[B41] Van de Putte G, Kristensen GB, Baekelandt M, Lie AK, Holm R (2004). E-cadherin and catenins in early squamous cervical carcinoma. Gynecol Oncol.

[B42] Jeffers MD, Paxton J, Bolger B, Richmond JA, Kennedy JH, McNicol AM (1997). E-cadherin and integrin cell adhesion molecule expression in invasive and in situ carcinoma of the cervix. Gynecol Oncol.

[B43] Sun H, Liu X, Li M (2000). E-cadherin expression and its clinical significance in cervical cancer. Zhonghua Zhong Liu Za Zhi.

[B44] Imura J, Ichikawa K, Takeda J, Fujimori T (2001). Beta-catenin expression as a prognostic indicator in cervical adenocarcinoma. Int J Mol Med.

[B45] Behrens J, Vakaet L, Friis R, Winterhager E, Van Roy F, Mareel MM, Birchmeier W (1993). Loss of epithelial differentiation and gain of invasiveness correlates with tyrosine phosphorylation of the E-cadherin/beta-catenin complex in cells transformed with a temperature-sensitive v-SRC gene. J Cell Biol.

[B46] Aberle H, Schwartz H, Kemler R (1996). Cadherin-catenin complex: protein interactions and their implications for cadherin function. J Cell Biochem.

[B47] Shinohara A, Yokoyama Y, Wan X, Takahashi Y, Mori Y, Takami T, Shimokawa K, Tamaya T (2001). Cytoplasmic/nuclear expression without mutation of exon 3 of the beta-catenin gene is frequent in the development of the neoplasm of the uterine cervix. Gynecol Oncol.

[B48] Henshall S (2003). Tissue microarrays. J Mammary Gland Biol Neoplasia.

[B49] Hsu FD, Nielsen TO, Alkushi A, Dupuis B, Huntsman D, Liu CL, van de Rijn M, Gilks CB (2002). Tissue microarrays are an effective quality assurance tool for diagnostic immunohistochemistry. Mod Pathol.

[B50] Rimm DL, Camp RL, Charette LA, Costa J, Olsen DA, Reiss M (2001). Tissue microarray: a new technology for amplification of tissue resources. Cancer J.

[B51] van de Rijn M, Gilks CB (2004). Applications of microarrays to histopathology. Histopathology.

[B52] DiVito KA, Charette LA, Rimm DL, Camp RL (2004). Long-term preservation of antigenicity on tissue microarrays. Lab Invest.

